# The Impact of COVID-19 on Members of the Saudi Community: Perceptions and Expectations From the Pandemic

**DOI:** 10.7759/cureus.34638

**Published:** 2023-02-05

**Authors:** Alaa Albeyahi, Bader Abou Shaar, Mohamed S Mohamed, Baraa Alghalyini

**Affiliations:** 1 Family and Community Medicine, College of Medicine, Alfaisal University, Riyadh, SAU; 2 Public Health, Saudi Ministry of Health, Riyadh, SAU; 3 Emergency Medicine, King Faisal Specialist Hospital and Research Centre, Riyadh, SAU; 4 Family and Community Medicine, University of Toronto, Toronto, CAN

**Keywords:** curfew, psychological impact of covid-19, pandemic perceptions, saudi community, covid-19 impact, covid-19 pandemic, community medicine & public health, saudi arabia, community prediction, world pandemic

## Abstract

Background and purpose

Community individuals and sectors worldwide, including health, security, economy, education, and occupation, are being challenged to confront the coronavirus disease 2019 (COVID-19) pandemic. The deadly virus originated in Wuhan city in China and spread globally to other countries on account of its rapid mode of transmission. Solidarity and cooperation were vital elements in mitigating the COVID-19 pandemic all around the globe. Actions of solidarity among countries included gathering the world's leading experts to discuss the latest research and innovation while working to promote knowledge and empowerment across the communities. The purpose of this study was to explore the influence of the COVID-19 pandemic on different aspects of the Saudi community, including health, education, finance, lifestyle, and others. We also wanted to identify the perceptions of the general Saudi population regarding the impact of the pandemic and its long-term effects.

Methodology

This cross-sectional study was conducted from March 2020 to February 2021 among individuals across the Kingdom of Saudi Arabia. The online self-developed survey was disseminated to thousands of individuals in the Saudi community and yielded 920 responses.

Results

Roughly 49% of the studied participants postponed their dental and cosmetic center appointments, and 31% reported delayed periodic health appointments in hospitals and primary healthcare centers. Around 64% reported missing hearing "Tarawih/Qiyam" Islamic prayers. Furthermore, 38% of the study respondents reported being anxious and stressed, 23% reported having sleeping disorders, and 16% wanted to be isolated from the community. On the other hand, the COVID-19 pandemic helped approximately 65% of the studied participants to abstain from ordering from restaurants and cafes. Additionally, 63% of them reported gaining new skills or behaviors during the pandemic. Many participants (54%) thought that after the curfew recession, they will face financial challenges while 44% assumed that life will not return to what it used to be.

Conclusion

The COVID-19 pandemic has had a multidimensional impact on Saudi society, which has affected both individuals and the community as a whole. Some of the short-term perceived impacts involved interruption in the provision of health care, poor mental well-being, financial hardship, challenges related to homeschooling and working from home, and the inability to fulfill spiritual needs. On the bright side, community individuals demonstrated the ability to learn and develop during the pandemic by striving to acquire knowledge and new skills.

## Introduction

The coronavirus disease 2019 (COVID-19) originated in Wuhan city in China and spread globally to other countries on account of its rapid mode of transmission [1]. COVID-19 was declared a pandemic within almost two months of the first reported case, and more than 213 countries worldwide were affected [2]. Community individuals and sectors worldwide, including health, security, economy, education, and occupation, were challenged to confront this pandemic.

The COVID-19 pandemic has had its social and economic consequences on people's lives. Although working from a distance was beneficial for a high percentage of people either occupationally or economically, low-wage and low-skill employees were more severely affected during this pandemic according to their limited abilities and are more likely to suffer from high turnover rates and poor working conditions [3]. Moreover, this pandemic has affected education, and many institutions converted to online learning in many countries across the world. Students suffered from decreased socialization due to the absence of on-campus activity, which affected their social interaction in group projects, technical and monetary issues, and response time. All of these were difficulties faced by students through online learning during the pandemic curfew [4]. Likewise, people suffered from different mental health problems such as stress, anxiety, depression, insomnia, denial, anger, and fear [5].

Although it was challenging to combat the COVID-19 pandemic in different aspects, the Saudi government, represented by its authorities, invested heavily in monitoring the COVID-19 situation through social distancing measures and mass gatherings' risk assessment by applying the following actions for the first time in decades: suspension of Umrah seasons, transitioning to learning and work platforms "from a distance", canceling a number of sports, cultural, and entertaining events, imposing national and international travel bans, and applying partial and total curfews depending on the pandemic situation [6].

During pandemics, people experience stressful circumstances that could affect their overall well-being. A recent study states that the recommended protective measures against COVID-19, such as self-isolation and home quarantine, have negative effects on the individual’s mental health [7]. A systematic review and meta-analysis reported that many patients who recovered from COVID-19 have developed persistent symptoms of depression and anxiety [8]. The lockdown along with the curfew law and fear of infection resulted in a psychological dilemma that is yet to be studied. The psychological impact of COVID-19 on people was evident as studies have reported the development of different types of mental health problems such as anxiety, depression, insomnia, denial, anger, and fear [5].

Social distancing plays an essential role in reducing the risk of infection and disease transmission between people [9]. Additionally, greeting among the inhabitants of the Mediterranean region is deemed very critical to establishing an interpersonal relationship or starting a social conversation [10,11]. It also takes several close contact approaches such as handshakes, cheek-to-cheek kissing, or hugging, especially in the Mediterranean region [10]. Moreover, a study demonstrated that Arabs tend to keep a closer personal distance during their communications to an extent considered uncomfortable by westerners [12]. Therefore, due to the cultural norm characterized by the prevalence of strong family ties and frequent social gatherings, it was challenging for people from the Saudi culture to adapt to the new distancing measures [6,13].

An important aspect of Saudi culture is practicing religion. Saudi Arabia is home to two of Islam’s holiest sites (i.e., Mecca and Medina). For people residing within the Kingdom of Saudi Arabia, religion and religious practices are an integral part of life and existence. On the 4th of March, a temporary unprecedented suspension of the Umrah pilgrimage for both citizens and residents of the Kingdom was declared. For the first time in decades, the pilgrimage “Umrah” to the Holy Mosque in Mecca was suspended as a precautionary measure to combat the virus despite the potential economic and political consequences. It was, therefore, challenging and emotionally straining for the Saudi community to withstand the social and spiritual challenges during the Ramadan and Eid periods [6].

The purpose of this study was to explore the influence of the COVID-19 pandemic on different aspects of the Saudi community, including health, education, finance, lifestyle, and others. We also wanted to identify the perceptions of the general Saudi population regarding the impact of the pandemic and its long-term effects.

## Materials and methods

This cross-sectional study was conducted from March 2020 to February 2021 among individuals across the Kingdom. Simple random sampling was applied according to the reported population size in the Saudi Arabia national census in 2019, which was 34,218,169 [14]. OpenEpi software version 3 was used to calculate the sample size [15].

All individuals living in Saudi Arabia aged 15 years old and above were included. All Saudi and non-Saudi individuals not currently living in Saudi Arabia and individuals below 15 years of age were excluded.

SurveyMonkey (Momentive Inc., Waterford, NY) was used to create the questionnaire [16]. It comprised four sections, with 23 (open and close-ended) questions, including demographic characteristics and questions about the influence of the crisis on religion, income, health, future perceptions, and expectations. These questions included branching logic to minimize the length of the questionnaire and were provided in both Arabic and English. Informed consent was taken after explaining the research purpose and ethical considerations. Alfaisal University's Institutional Review Board approved this study (IRB: 20035).

The self-developed online questionnaire was disseminated to individuals in the Saudi community through social media (WhatsApp, Twitter, and Snapchat) in May 2020 before the curfew was lifted. A pilot study was done among 20 participants: 10 for each language (Arabic and English) before disseminating the questionnaire to the public. To increase the response rate, participants were invited to enter a draw for prizes of 10 gift vouchers chosen randomly.

Data were analyzed using Microsoft Excel version 2016 (Microsoft Corporation, Redmond, WA) and then analyzed using the Jamovi software (version 1.0.8.0) [17,18]. Estimates were reported using frequencies and proportions, while significance was tested using the chi-square test. Statistical significance was determined based on a p-value of <0.05, and results were determined as highly statistically significant for a p-value of less than <0.01.

## Results

This study included 920 participants who fully completed the questionnaire and the demographic data are shown in Table 1.

**Table 1:** The demographic characteristics of the studied participants.

Demographic characteristics		Count	%
Age	15-25 years old	226	24.6%
	26-36 years old	302	32.8%
	37-47 years old	221	24.0%
	48-58 years old	132	14.3%
	59 years old and above	39	4.2%
	Total	920	100.0%
Gender	Male	319	34.7%
	Female	601	65.3%
	Total	920	100.0%
Nationality	Saudi	877	95.3%
	Non-Saudi	43	4.7%
	Total	920	100.0%
Kingdom region	Central region	722	78.5%
	Southern region	64	7.0%
	Eastern region	49	5.3%
	Northern region	14	1.5%
	Western region	71	7.7%
	Total	920	100.0%
Marital status	Married	512	55.7%
	Single	408	44.3%
	Total	920	100.0%
Monthly income	Less than 5,000 Saudi riyals	177	19.2%
	5,000-10,000 Saudi riyals	212	23.0%
	11,000-15,000 Saudi riyals	162	17.6%
	More than 15,000 Saudi riyals	215	23.4%
	No monthly income	154	16.7%
	Total	920	100.0%
Education	High-school diploma	154	16.7%
	Undergraduate degree	586	63.7%
	Postgraduate degree	180	19.6%
	Total	920	100.0%
Employment sector	Governmental	402	437%
	Military	30	3.3%
	Private	171	18.6%
	Employer	11	1.2%
	Student	151	16.4%
	Unemployed/housewife	108	11.7%
	Retired	22	2.4%
	Others	25	2.7%
	Total	920	100.0%

Roughly 49% of the studied participants had their dental and cosmetic appointments postponed, while 31% had their periodic appointments in hospitals or primary healthcare centers postponed. Furthermore, 25% of participants indicated that e-learning/distance working saved their time, effort, and money. In contrast, 24% said that they had to work all day due to online work/e-learning. Spiritually, 64% yearned for hearing the call to “Tarawih” and “Qiyam” prayers. Many participants (44%) missed praying at mosques and 43% missed having the ability to perform Umrah.

The COVID-19 pandemic helped 65% of the participants stop ordering take-out from restaurants and cafes, and 63% of them stopped or cut back on going to malls and markets. While 49% of them said that the pandemic helped reduce their financial burden by stopping or significantly reducing regular gatherings with family and friends. Interestingly, 29% stated that they stopped or reduced their online shopping.

On the topic of psychological health, 44% of the total participants stated that they were not affected psychologically or mentally by the pandemic. However, 38% of them reported being anxious and stressed, 23% reported having sleeping disorders, and 16% of them wanted to be isolated from the community. With regards to their social life, 44% of the studied participants said that their relationship with their family improved during the pandemic, while 38% stated that their relationships with friends had improved. Approximately 49% completely stopped or reduced regular gatherings with family and friends.

As shown in Table 2, the majority (63%) of the studied participants reported gaining new skills or behaviors during the pandemic. Additionally, around 65.5% of males gained new skills and behaviors during the pandemic compared to 61.9% of females but no statistically significant association was indicated. Females reported significantly higher habits of daily reading/memorizing/“Khatam” of the Holy Quran (p = 0.025) during the pandemic compared to males. Of the females, 8% reported developing cooking or professional cooking as a new habit, and it was the most common new skill developed across all habits for females.

**Table 2:** New skills and behaviors acquired during the pandemic among participants based on gender. * Statistically significant.

New skills acquired during the pandemic		Gender						Chi-square test
		Male		Female		Total		
		N	%	N	%	N	%	P-value
Did you gain any new skills or behaviors during the pandemic?	Yes	209	65.5%	372	61.9%	581	63.2%	.279
	No	110	34.5%	229	38.1%	339	36.8%	
Daily reading/memorization/“Khatam” of the Holy Quran	Yes	2	0.6%	17	2.8%	19	2.1%	.025*
	No	317	99.4%	584	97.2%	901	97.9%	
Adherence to religious duties (more time to worship/praying on time/praying in congregation)	Yes	8	2.5%	12	2.0%	20	2.2%	.613
	No	311	97.5%	589	98.0%	900	97.8%	
Reading/reading improvement/books in different languages	Yes	15	4.7%	23	3.8%	38	4.1%	.525
	No	304	95.3%	578	96.2%	882	95.9%	
Household chores (cleaning/planting/decoration/maintenance/management)	Yes	9	2.8%	12	2.0%	21	2.3%	.425
	No	310	97.2%	589	98.0%	899	97.7%	
Cooking casually/professionally	Yes	12	3.8%	48	8.0%	60	6.5%	.014*
	No	307	96.2%	553	92.0%	860	93.5%	
Acquiring healthy habits (workout/running/food/sleeping/meditation/yoga)	Yes	8	2.5%	25	4.2%	33	3.6%	.200
	No	311	97.5%	576	95.8%	887	96.4%	
Financial skills (planning/capital market learning/saving)	Yes	4	1.3%	3	0.5%	7	0.8%	.210
	No	315	98.7%	598	99.5%	913	99.2%	
Time management	Yes	4	1.3%	10	1.7%	14	1.5%	.629
	No	315	98.7%	591	98.3%	906	98.5%	
Spending leisure time (learning new knowledge/cultures/hobbies/skills)	Yes	18	5.6%	46	7.7%	64	7.0%	.254
	No	301	94.4%	555	92.3%	856	93.0%	
Art skills (painting/handcrafting/decoupage/art/gift-wrapping/recycling/sewing/music)	Yes	3	0.9%	19	3.2%	22	2.4%	.036*
	No	316	99.1%	582	96.8%	898	97.6%	
Computer skills (programming/technological/digital skills/gaming/programming/designing/development)	Yes	5	1.6%	7	1.2%	12	1.3%	.608
	No	314	98.4%	594	98.8%	908	98.7%	
Being close to/taking care of my parents/family/children	Yes	8	2.5%	12	2.0%	20	2.2%	.613
	No	311	97.5%	589	98.0%	900	97.8%	
Learning sterilization/caution/prevention measures	Yes	5	1.6%	9	1.5%	14	1.5%	.934
	No	314	98.4%	592	98.5%	906	98.5%	
Dealing with crisis/problems/stress/marital problems	Yes	2	0.6%	4	0.7%	6	0.7%	.945
	No	317	99.4%	597	99.3%	914	99.3%	
Adapting to and enjoying the available resources	Yes	4	1.3%	2	0.3%	6	0.7%	.099
	No	315	98.7%	599	99.7%	914	99.3%	
Patience	Yes	10	3.1%	15	2.5%	25	2.7%	.571
	No	309	96.9%	586	97.5%	895	97.3%	
Commitment/responsibility/awareness/cautions	Yes	6	1.9%	4	0.7%	10	1.1%	.091
	No	313	98.1%	597	99.3%	910	98.9%	
Prioritization/focusing on my own goals	Yes	1	0.3%	5	0.8%	6	0.7%	.352
	No	318	99.7%	596	99.2%	914	99.3%	
Self-development (willingness/reassessing/self-care/self-control/anger control/self-dependency/training-to-be-focused/thinking intensively)	Yes	8	2.5%	19	3.2%	27	2.9%	.576
	No	311	97.5%	582	96.8%	893	97.1%	

Many participants (54%) thought that after the pandemic's recession, they will face financial challenges and 44% assumed that life will not return to what it used to be. Around 33% anticipated occupational challenges such as a deficiency in the job market. Moreover, 15% predicted the re-occurrence of the COVID-19 pandemic or other outbreaks, and 61% of them expect themselves to become more appreciative of things after the resolution of the pandemic.

## Discussion

The Saudi 2030 Vision represented by the National Transformation Program plays an important role in enhancing the healthcare system and facilitating access to health care by integrating technology and establishing universal health coverage [19]. Saudi Arabia made a huge effort to ensure dispensing/delivering medications to all patients to their homes during the curfew. Despite the government's efforts, the COVID-19 outbreak resulted in delayed healthcare appointments with a majority being dental and cosmetic center appointments. This might be due to the fear of spreading or catching the infection through the mouth/nose where COVID-19 can be easily transmitted in dental clinics. Researchers at a private dental clinic in Brazil found that many patients already undergoing treatment would rather go to their dental appointments due to fear of having their treatments delayed, while patients under no treatment reported that they would not attend their appointments or would visit only in case of an emergency [20]. Also, postponing or cancellation of cosmetic/dermatology center appointments was significantly higher among females in comparison with males (p < 0.001). One study found that postponing non-essential appointments by dermatology practitioners increased from 35.5% to 79.4% to 95.6% during the pandemic [21]. This could be attributed to the people's fear of going to hospitals that admitted COVID-19-positive cases or because of hospital-related quarantine measures. Similarly, 35.2% of Italian patients have canceled their appointments because of COVID-19 fears during the lockdown, and 29.4% of them after the lockdown [22].

Hospital and primary care periodic checkup appointments were reportedly postponed by approximately 31% of individuals. This finding is comparable to that observed by Al-Kuwari et al., who revealed a 40% decline in the number of well-baby and immunization service appointments utilized between March and May 2020 compared to the same period in 2019 [23]. To combat this decline, the Saudi Ministry of Health encouraged the Saudi community to avoid delaying children's vaccination appointments through the "#Do_Not_Delay_It" Twitter hashtag [24]. Although some participants postponed their medical appointments, others reported using technology (e.g., video conference and "Mawid" mobile application) to follow up with their doctors or searching the internet for medical advice [25].

The studied participants reported that e-learning and working from home helped them to save more time, effort, and money that was previously spent in preparing (e.g., meals and transit time) to go to school, university, or work. Similar findings were reported by Hussein et al., who reported that online learning had many positive aspects, including saving time in getting ready and commuting to school, eliminating transportation costs, reducing the risk of car accidents, and increasing students' self-esteem [26]. "Working all day" during the pandemic was reported by 24% of the participants, likely due to the continuous work/studying lifestyle adapted due to the pandemic. In line with what Green et al. found, working from home led to intensification, extended working hours, and difficulty to maintain a proper work-life balance [27].

Difficulties in communication and understanding between students and teachers, or between employers and employees or co-workers, were reported by study participants. This may be due to the abrupt shift to technology for the entire population, causing this challenge to arise. Globally, e-learning had been challenging for teachers due to the lack of training in online teaching skills, tedious online lesson preparations, lack of technical support, and educational platform overload [28]. E-learning was equally challenging for students due to the lack of an appropriate learning attitude, lacking learning materials, self-discipline incapability, and inadequate environments for studying at some homes [29].

The pandemic affected the spirituality of the Saudi community. Many participants expressed how they missed performing Islamic rituals during the month of Ramadan, including praying Taraweeh, visiting the holy mosques in Makkah and Medina to perform Umrah (an Islamic minor pilgrimage), and seclusion in the mosque. On the other hand, many participants were positively affected and became closer to Allah (God) during the curfew and tended to practice the following: maintain their prayers, practice "Witr" prayer, read the Holy Quran frequently, increased their spirituality and faith, and were satisfied and content with the simplest things. Thomas et al. found a statistically significant association between positive religious coping among Muslims when compared to Christians [30].

The community's perception of the influence of precautionary measures on their finances has been variable. On one hand, some participants managed to reduce spending on meals and instead prepared home-cooked meals because they were unable to dine out in restaurants during the curfew. Many found that cooking at home improved bonding with immediate family members. On the other hand, others were relying on online delivery from restaurants, cafes, supermarkets, and other shops instead of going to the malls or other places even during the partial curfew. Restrictions placed by the Saudi government on large familial/social gatherings also limited the financial burden on individuals, especially after canceling the "cost-of-living allowance" funds by the government in June 2020 and increasing the value-added tax (VAT) from 5% to 15% in July 2020 [31]. Participants did, however, express frustration from expenses related to purchasing pandemic-related requirements such as personal protective equipment, including face masks and hand/surfaces sanitizers, as well as mandatory delivery fees due to the curfew.

A minority of our participants anticipated occupational challenges and incurring debts during the stagnant job markets throughout the curfew. This is similar to what was reported by an article that found that over 20 million employees had lost their jobs by April 6th, 2020, and those who lost their jobs were not interested in looking for new ones [32]. The percentage of participants in the labor force also reportedly declined by 7% [32]. Considering these financial burdens on the Saudi community, the Saudi government declared an initiative to extend support to the affected Saudi workers affected by the repercussions of COVID-19 in private sector establishments [33].

Our study reflected a diverse and broad range of consequences the pandemic had on people's lifestyles and daily activities. Many, however, found the pandemic to be a great opportunity to adopt a healthier lifestyle by starting to exercise and eat healthier food. This could be explained by the flexibility offered by working from home and a subsequent decrease in social commitments because of the lockdown. In contrast, a study done in Uganda reported an unhealthy lifestyle amidst the pandemic [34]. On the other hand, some of the participants reported that the pandemic weakened their commitment to do physical exercises or diet and missed doing exercises/attending gyms because of the total curfew and the cancelations of gym or food subscriptions. This supports the study that was done to measure the changes in physical activity among the Saudi population before and during the curfew, which found a decrease in physical activity for most participants and an increase in sedentary lifestyles during curfews [35].

Psychologically, many of the studied participants reported that the pandemic did not affect their psychological/mental health while many of them became anxious, stressed, experienced insomnia, and had reduced productivity because of the unexpected and unusual situation in the community. These findings are in line with Alkhamees et al., who found that the COVID-19 pandemic was highly associated with stress, anxiety, and depression, especially among healthcare workers, students, and females [36]. It was also reported that adhering to preventive measures was a protective effect against these psychiatric disorders [36]. Another study reported that the COVID-19 pandemic was associated with significant mental health hazards in the Saudi population [37]. Additionally, recent articles found that the COVID-19 lockdown was associated with disorders in sleeping schedules, quantity, and quality of sleep at night [38,39]. Many clinical trials have already begun testing therapeutic management options for persisting symptoms of COVID-19 or post-acute COVID-19 syndrome [40].

Participants reported different approaches to limit pandemic-induced stress and anxiety. Some decided to isolate themselves from social media and daily COVID-19 conferences to avoid hearing about the rise in infected, hospitalized, or deceased cases. This is concordant with Farooq et al., who revealed that cyberchondria and information overload were significantly associated with individuals' tendency to self-isolate and feel threatened [38]. In contrast, some participants appreciated the pandemic lockdown for offering them an opportunity to meditate and self-reflect. This finding is supported by Al-Qahtani et al., who reported that 65.5% of the Saudi participants felt that social distancing makes them feel comfortable and peacefully minded [41]. Another article revealed that psychological distress was associated with underlying misconceptions about the COVID-19 pandemic in Saudi Arabia [42].

Some participants reported reducing the number of gatherings with family and friends because of the pandemic. One of the most important reasons is that families living in different cities or countries were restricted from traveling during the curfew and were unable to enjoy celebrating Islamic holidays together (e.g., Ramadan and Eid). On the other hand, others, especially males, were happy to spend more time with their immediate families because of the pandemic lockdown. These findings are consistent with an article that found that the COVID-19 lockdown positively affected familial relationships [43].

The COVID-19 pandemic offered many of the studied participants the opportunity to gain new skills or behaviors during the curfew. Utilizing leisure time with online training courses was the most frequently gained skill. More people started to watch the news, videos, and podcasts during the pandemic. Both professional and leisure cooking were considered a newly gained hobby for many individuals during the pandemic. Community individuals found it better and safer to cook their meals than bring them from outside the home in the middle of the crisis. This is similar to a study that reported bread baking as an activity that was shared through social media during the curfew [44].

Moreover, several participants developed their own businesses during the pandemic, which allowed people to have free time to think and build for their future. As reported by Awan, by the end of the second quarter of 2020, roughly 571.7 thousand small and medium enterprises were established in Saudi Arabia with 2% of them being new establishments entering the economy sector [45]. Many participants gained the skills of cosmetology and shaving during the pandemic curfews as every shop was either closed or suspended. Other interesting skills gained included decision-making, etiquette, increasing social unity and affinity through understanding others, taking care of them, and not judging them for any reasons like before. All in all, the COVID-19 pandemic gave us free lessons and life experiences that will last forever.

The majority of the studied participants indicated financial challenges (budget declining) as future perception after the total curfew lifting. This may be reflected by the amount of entertainment or healthcare facilities closure, including some restaurants and cafes, cinemas, playground areas, gyms, and private cosmetic or dental centers. However, it was expected in the fiscal year of 2020 that the budget deficit will increase to Saudi riyal (SAR) 298 billion, and it was aimed to decrease the budget deficit to SAR 141 billion or 4.9% of GDP in the fiscal year of 2021 [46].

The overburden of the Saudi health system is predicted by most of the studied participants. This may result from fears of healthcare workers' eventual exhaustion due to the pandemic. This is consistent with Skoda et al., who found that nurses were more likely to be psychologically overwhelmed compared to other healthcare professionals [47]. Another article reported that the healthcare system was so severely affected by COVID-19 worldwide that it led to the accidental disruption of the proper delivery of care for other chronic or communicable diseases [48].

Many participants predicted the recurrence/reemergence of the COVID-19 pandemic or other future pandemics after the total curfew lifting. This finding is concordant with an article that reported that this COVID-19 pandemic pattern has a high probability of developing multiple waves and therefore greater preparedness for upcoming waves is required [49]. Furthermore, enhancing communication and social synergy among community individuals after the COVID-19 pandemic is over was encouraged by many of the studied participants. This supports findings that concluded that the COVID-19 pandemic and its social distancing can never replace direct and physical contact with friends and its associated feelings of loneliness [41]. Finally, many study participants reported being more appreciative of the blessings they have, including governmental efforts, time, jobs, schools, universities, and relationships with family and friends.

Strengths

Our study gathered intriguing findings about the Saudi community, to which we were oblivious. This would be the first study of its kind to address the pandemic's psychological, financial, and spiritual impact on the Saudi community and their way of living. Our goal was to understand how individuals were affected during the pandemic, hoping this will help prepare communities and individuals for anticipated future waves and pandemics.

Limitations

Because this study was limited to the curfew period, we had to disseminate the survey for a limited time during the lockdown period to confirm the attainment of accurate responses. Even with offering the questionnaire in the English language, our study was deficient in responses from expatriate employers. Recall bias and question misinterpretation may have occurred because our data were collected through a self-administered questionnaire. In an attempt to avoid this, the survey underwent a pilot phase to assess for clarity and quality of the questions prior to distribution. The relatively small response rate was also another limitation. Despite enhancing the dissemination of the questionnaire by sharing it on different social media platforms (WhatsApp, Twitter, and Snapchat), a high response rate was difficult to achieve.

## Conclusions

The COVID-19 pandemic had a multidimensional impact on Saudi society, affecting both individuals and the community as a whole. Some of the short-term perceived impacts involved interruption in the provision of health care, poor mental well-being, financial hardship, challenges related to homeschooling and working from home, and the inability to fulfill spiritual needs. Additionally, the Saudi community predicted that after the recession of the pandemic and imposed curfew restrictions, life would not be the same as before the pandemic, especially regarding work and education. Many respondents were optimistic about acquiring new skills and social networking.

The studied participants demonstrated the ability to learn and develop during the pandemic by gaining knowledge and skills, including sports, healthy habits, and starting new investments or businesses. These results can help us recommend further actions to be implemented to enhance the learning and development cycle in Saudi Arabia, such as involving startups and enhancement projects for community individuals to achieve their goals and contribute to Saudi Arabia’s vision for growth and success. Furthermore, we recommend civil engineers and décor designers construct modern and energetic home buildings with entertaining facilities and green areas to ease living through future pandemics.
